# Longitudinal association between psychological demands and burnout for employees experiencing a high versus a low degree of job resources

**DOI:** 10.1186/s12889-018-5778-x

**Published:** 2018-07-25

**Authors:** Anna-Carin Fagerlind Ståhl, Christian Ståhl, Peter Smith

**Affiliations:** 1 Department of Medical and Health Sciences, Linköping University, Linköping, Sweden; 2 HELIX Competence Centre, Linköping University, Linköping, Sweden; 3 Institute for Work & Health, 481 University Ave, Suite 800, Toronto, ON M5G 2E9 Canada; 4 Dalla Lana School of Public Health, University of Toronto, Toronto, Canada; 5 Department of Epidemiology and Preventive Medicine, Monash University, Melbourne, Australia

**Keywords:** Work environment, Stress, Social capital, Sick leave, Work disability, Prevention, Sweden

## Abstract

**Background:**

Exhaustion and burnout are common causes for sickness absence. This study examines the relationship between psychological demands and burnout over time, and if environmental support modifies the longitudinal relationship between psychological demands and burnout at baseline, with burnout measured 2 years subsequently.

**Methods:**

A questionnaire was sent to employees in seven Swedish organizations in 2010–2012 with follow-up after 2 years, *n* = 1722 responded (64%). Linear regressions were used to examine the associations between burnout and psychological demands at baseline and burnout at follow-up. Stratified regression models examined if relationships between burnout and psychological demands at baseline on burnout at follow-up differed for employees in supportive versus unsupportive work environments.

**Results:**

Burnout and psychological demands at baseline were associated with burnout at follow-up, after adjustment for study covariates. No significant differences were observed between estimates for psychological demands and burnout among respondents in supportive work environments versus those in unsupportive work environments.

**Conclusions:**

This study shows that high demands are associated with greater risk of burnout, regardless of level of other work supports. This has implications for prevention of sick leave due to burnout and for rehabilitation, where demands such as work pace, workload and conflicting demands at work may need to be reduced.

## Background

Emotional exhaustion and reactions to severe stress are at the present time the dominating causes for sickness absence in Sweden [1]. Exhaustion, or feelings of fatigue and being worn out, is often considered the core dimension of burnout [2, 3], a work-related syndrome that according to some researchers also include feelings of cynicism and a sense of reduced efficacy and accomplishment [3, 4]. Burnout is in many cases chronic in its nature [5, 6] and has far-reaching consequences for the individual as well as society. Besides prolonged emotional distress for the individual it implies increased long-term risks for depression and other mental health problems, for medication [5–7] as well as hospitalization due to mental as well as cardiovascular disorders [6].

The relationship between low job control, low social support, and high psychological demands with increased burnout over time are well established [8, 9]. The Demand-Control model identifies psychological demands and control as central aspects of work in relation to the development of strain [10, 11]. Subsequent development of the demand-control model also included instrumental and emotional support from colleagues and supervisors as a third dimension [10, 12]. Similarly, the Job Demands-Resources model explains how exhaustion and burnout occur in the presence of high job demands and absence of job resources [13]. According to the Job Demands-Resources model, psychosocial work conditions can, regardless of the job, be divided into two dimensions: job demands or aspects of the job that require sustained effort and therefore are associated with certain physiological or psychological costs, and job resources or aspects that reduce the negative effect of demands, are functional in achieving goals and foster development [14]. Both the Demand-Control and Job Demands-Resources model assume that other aspects of the work environment may buffer the health-impairing effect of job demands. For example, according to the demand-control model [11], a high degree of skill use and autonomy in decision-making concerning work tasks will give the individual control over which skill to use to accomplish a task. Enabling a wider range of solutions and strategies to be utilized to deal with work demands will in turn counter the development of strain. In the Job Demands-Resources model, job resources are assumed to buffer the negative impact of job demands on strain and burnout, and hinder the development of exhaustion [15].

The additive effect of demands, decision latitude, and social support on burnout has been well investigated. Previous studies have observed that a high degree of decision latitude as well as helpful social interactions at work from colleagues and supervisors reduced strain and exhaustion [16], and the perceived fairness and justice in work groups were important for whether employees develop symptoms of burnout or not [17]. However, it is still unclear whether decision latitude and other job resources have the potential to buffer or attenuate the effect of high psychological demands on adverse health such as burnout. Few studies have longitudinally investigated (and found support for) the buffer hypothesis [18–21]. Associations found in cross-sectional studies are more prone to issues of reverse causation between exposures and outcomes [22, 23]. In addition, measurements of supports at only one point in time may be less accurate than if taken over multiple time points [11, 24, 25]. To better understand the relationship between psychological demands and burnout, and the moderating impact of workplace supports requires a longitudinal design.

The objective of this paper is to examine whether the impact of baseline psychological demands and burnout on future burnout differs for employees with high job resources compared to those with low resources. Based on the demand-control and demand-resources models we hypothesize that the effects of psychological demands and burnout on future burnout should be weaker among employees who have high resources (high decision latitude and high workplace social capital), compared to employees who have low job resources.

## Methods

### Material

**Fig. 1:**
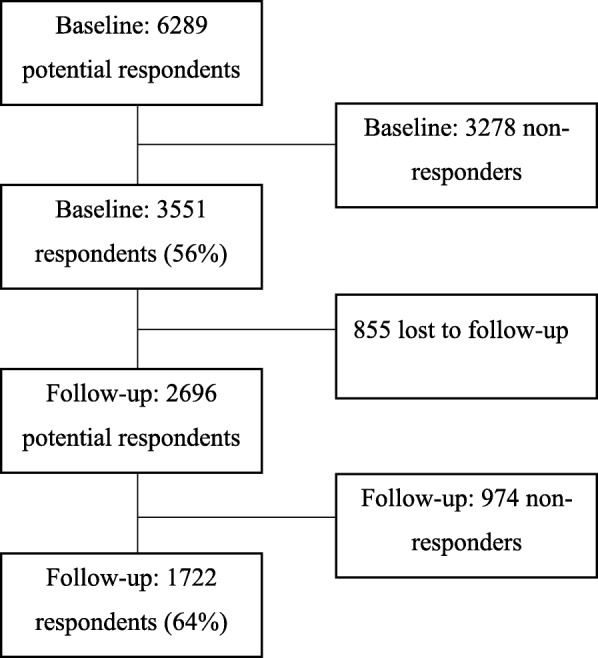
Flow-chart of the data collection

A questionnaire was sent to 6289 employees in seven Swedish organizations in 2010–2012 with a follow-up after 2 years. Inclusion criteria were being employed in one of the participating organizations and understanding Swedish. Prior to the distribution of questionnaires, organizational schedules and lists of employees containing names, age and gender were retrieved from the organizations. For the follow-up, organizational schedules and lists of employees were again collected and manually checked for changes, where the follow-up questionnaires were only sent to employees who had participated at baseline. Employees in the organizations received a questionnaire on paper or electronically. The paper questionnaires were distributed in individually addressed and closed envelopes which included a coded survey, an informative letter concerning the project, and a pre-stamped response envelope. The electronic version was sent along with an informative letter to employees’ personal work e-mail. Employees were allowed to fill in the questionnaire during working hours. In total, *n* = 3551 responded at baseline (56%). At follow-up, 2696 of the respondents at baseline were still employed and received a second questionnaire to which *n* = 1722 responded (64%). In this final longitudinal sample, *n* = 295 were employed in a private production company, *n* = 183 in a private care company, *n* = 477 in a municipal organization including several different occupations, *n* = 121 in a different municipal care unit, *n* = 68 in a public care organization, *n* = 322 in a government authority, and *n* = 256 in a government organization. The mean age was 49 years at follow-up (standard deviation = 10.20 years), 66% were women and the majority had high school/vocational school education. A flow-chart of the data collection is shown in Fig. 1.

### Measures

Outcome: The main outcome for this study was burnout measured at the time two survey. Burnout was measured at both time points using the generic subscale “Personal burnout” from The Copenhagen Burnout Inventory [2]. It consists of six items concerning physical and psychological fatigue (e.g. How often are you emotionally exhausted? How often do you feel worn out?). Internal consistency: Cronbach’s α .88. Reponses were standardized into a scale from 2 to 10, with higher scores indicating more burnout.

Main predictors: The main predictors were job psychological demands and burnout measured at the time one survey. The measurement of burnout is described above. Psychological demands was measured with The Swedish Demand Control Questionnaire [11, 26]. Five items measure psychological demands (e.g. Does your work demand that you work very hard? Is there enough time to perform work tasks?) Answers were given on a four-point Likert scale. Internal consistency for psychological demands: Cronbach’s α .79. Responses were standardized into a scale from 2 to 10, with higher scores indicating higher psychological demands.

Potential moderating variables: We used two measures to define supportive work environments. These were decision latitude and social capital. Decision latitude was measured with The Swedish Demand Control Questionnaire [11, 26], containing six questions (e.g. Do you have the opportunity to decide for yourself how to carry out your work? Do you have the opportunity to learn new things in your work?). Answers for decision latitude were given on a four-point Likert scale, with internal consistency of α .68. Social capital at work refers to the degree to which connections between individuals in the workgroup are trusting and enables collaboration and information sharing [27, 28], and was measured by eight items (e.g. Members of the work unit build on each other’s ideas in order to achieve the best possible outcome; People keep each other informed about work-related issues in the work unit). Answers were given on a five-point Likert scale. The internal consistency for social capital was Cronbach’s α .90. Responses for both dimensions were standardized into a scale from 2 to 10, with higher scores indicating higher decision latitude and social support.

To define supportive work environments over the study period we used both time one and time two measures of social capital and decision latitude, to reduce measurement error from only using one time point. Decision latitude and social capital at baseline and follow-up were dichotomized into high and low based on a median split approach. Based on categories and baseline and follow-up respondents were then grouped into the following four categories for each resource: consistently high (high at both time points); decreasing (high at baseline, low at follow-up); increasing (low at baseline, high at follow-up); and consistently low (low at both time points).

Covariates: Potential confounders for regression models included organization, gender, age (continuous), level of education, marital status and having children living at home.

### Analysis

Initial descriptive analyses examined the distribution of the sample across our main outcome, independent variable, moderating variables and covariates.

Linear regressions were used to examine the associations between burnout and psychological demands at baseline and burnout at follow-up. Initial analyses investigated how psychological demands and burnout at baseline were associated with burnout at follow-up, adjusted for decision latitude, social capital at work and demographics. Additional models examined if there were differences in the relationship between psychological demands and burnout at baseline on burnout at follow-up for employees who experienced a high versus a low degree of job resources over the follow-up period. For these models, we restricted our analyses to respondents who had either supportive environments at both time points (based on measures of social capital and decision latitude) or non-supportive environments at both time points (*N* = 1163 for analyses using decision latitude and *N* = 1079 for analyses using social capital). The restriction of the sample in this way was to better ensure the comparison of effects was between respondents with consistently high and consistently low job resources, given the increased accuracy gained by the use of two time points [25].

We examined differences in two ways. We first ran models which included an interaction term between psychological demands and burnout, and high/low resources (based on decision latitude and social capital scores at time 1 and time 2). Separate models were run for each interaction term, with four models being run in total (psychological demands and high/low decision latitude; psychological demands and high/low social capital; burnout and high/low decision latitude; and burnout and high/low social capital) To compliment these analyses we also ran a series of models, stratified by level of resources, to examine the relationships between baseline burnout and psychological demands on time two burnout for respondents with high and low decision latitude and social capital environments. All analyses were conducted using SAS V 9.3.

### Drop-outs and missing data analysis

Non-respondents at baseline were older (46 years) than respondents (45 years) (*p* = .013). There was no difference concerning gender. Compared to those respondents who responded to both surveys, those who did not respond to the follow-up had lower psychological demands (*p* = .028) and lower decision latitude (*p* = .022) and were younger (*p* = .006). There were no significant differences concerning burnout, gender or social capital at work. Given the high alpha estimates for the burnout, psychological demand, decision latitude and social capital scales, if respondents were missing responses on less than half of the questions in the scale, these values were imputed (up to two items for burnout, psychological demands and decision latitude and up to three items for social capital) [29]. This resulted in 9 respondents having imputed values for some burnout items at time one and 4 respondents having imputed values for items at time two; 42 respondents with some imputed values for psychological demands at time one; 25 respondents with some imputed values for decision latitude at time one and 28 respondents at time two; and 49 respondents with some imputed values for social capital items at time one, and 33 at time two. Of the sample of 1722 respondents who responded to the follow-up survey, 107 respondents (6.2%) had missing information on more than half of the items assessing study exposures, or information on outcomes or covariates. The majority of these missing responses were missing values for age (*N* = 59, 55% of missing responses), with another 32 respondents missing information on more than half of the items for a given work exposure. This left a final analytical sample of 1615 respondents. A logistic regression analysis examined the probability of missing information on age across organization and demographic variables with complete information. This analysis observed male respondents and those who were single were more likely not to have reported age. No relationship was found between demographic factors or organizational factors and the likelihood of missing responses on work exposures.

## Results

Table 1 presents the distribution of the study variables in the final analytical sample. Focusing on our main variables of interest mean psychological demands at baseline were 6.90 (scale 2 to 10) with a standard deviation of 1.42. Mean levels of burnout were similar at both time one and time two. Approximately 29% of our sample (*N* = 482) were classified as having a supportive work environment based on decision latitude scores at both time points, while 32% (*N* = 521) had a supportive environment based on social capital scores at both time points. Conversely, 42% of the sample had a non-supportive environment based on decision latitude scores, and 35% had a non-supportive environment based on social capital scores.

**Table 1:** Distribution of main study variables in the final analytical study sample (*N* = 1615)

Continuous psychological variables (range 2 to 10)	Mean	std
Psychological Demands	6.90	1.42
Burnout (Time one)	4.88	1.42
Burnout (Time two)	4.89	1.46
Categorical variables	N	%
Change in decision latitude		
High both time points	482	29.8%
Increasing	213	13.2%
Decreasing	239	14.8%
Low both time points	681	42.2%
Change in social capital		
High both time points	521	32.3%
Increasing	271	16.8%
Decreasing	265	16.4%
Low both time points	558	34.6%
Age Group		
15 to 34 years	223	13.8%
35 to 54 years	912	56.5%
55+ years	480	29.7%
Gender		
Male	549	34.0%
Female	1066	66.0%
Education		
Primary school	116	7.2%
Vocational school	478	29.6%
High school	415	25.7%
University degree	606	37.5%
Organization		
Private production company	266	16.5%
Private care company	175	10.8%
Municipal organization	444	27.5%
Government authority	300	18.6%
Municipal care unit	114	7.1%
Public health care organization	68	4.2%
Government organization	248	15.4%

Table 2 presents the results of our multivariable linear regression models examining the relationship between burnout and psychological demands at baseline and burnout at follow-up. For each unit increase in burnout at baseline, burnout at follow-up increased by 0.60 points (*p* < .001). For each unit increase in psychological demands at baseline burnout at follow-up increased by 0.11 points (*p* < .001), taking into account burnout scores at time one and adjusted for decision latitude, social capital at work and demographics.

**Table 2:** Adjusted ordinary least-squares (OLS) regression estimates for burnout and psychological demands at baseline on burnout at follow-up (*N* = 1615)

	Burnout at follow-up		
	Unstandardized beta estimate	95% CI	*p*-value
Burnout baseline	0.60	0.56–0.64	< .001
Psychological demands	0.11	0.06–0.16	< .001

Model adjusted for gender, age (continuous), level of education, marital status, children, organization, decision latitude, and social capital (all measured at baseline) as well as both variables included in the Table. Adjusted R^2^ = 0.425

Table 3 presents the results of the stratified analyses for psychological demands on burnout at follow-up among respondents with high and low decision latitude and social capital, as well as the *p*-value for the interaction term from a combined model (presented in the far right column of the table). No significant differences were observed between regression estimates for psychological demands and burnout among respondents with high decision latitude and social capital compared to those with low (*p*-value for interaction across decision latitude groups = 0.94; *p*-value for interaction across social capital groups = 0.97).

**Table 3:** Adjusted OLS regression estimates for psychological demands at baseline on burnout at follow-up for groups of employees reporting a high versus a low degree of job resources

	High			Low			*p*-value for interaction^a^
Decision Latitude (*N* = 1163)							
	Unstandardized beta estimate	95% CI	*p*-value	Unstandardized beta estimate	95% CI	*p*-value	
Psychological demands	0.10	0.01–0.20	0.03	0.11	0.03–0.18	0.003	0.94
Social Capital (*N* = 1079)							
	Beta	95% CI	*p*-value	Beta	95% CI	*p*-value	
Psychological demands	0.07	−0.02 – 0.15	0.13	0.05	−0.04 – 0.13.	0.27	0.97

Model adjusted for burnout at baseline, gender, age (continuous), level of education, marital status, children, and organization. Decision latitude models additional adjusted for social capital and social capital models additionally adjusted for decision latitude

Adjusted R^2^ = 0.378 for model with high decision latitude; 0.474 for model with low decision latitude; 0.391 for model with high social capital and 0.452 for model with low social capital

^a^The p-value for the interaction represents to p-value associated with the interaction between psychological demands and high/low decision latitude, or social capital, from a combined model

Table 4 presents the relationship between burnout at baseline on burnout at follow-up for respondents with high and low decision latitude and social capital. Similar to table three, estimates from the models stratified by high and low resources (decision latitude on top and social capital on bottom) are presented in the left hand side of the table, with the *p*-value estimate for the interaction term between burnout and level of resources, from a complimentary model including respondents with both high and low resource levels, presented on the column on the far right. Slightly stronger regression estimates were observed in the relationship between baseline burnout and future burnout for employees with low decision latitude and low social capital, compared to those with high values (0.64 versus 0.58 for decision latitude; 0.67 versus 0.59 for social capital), however in neither case did these differences reach traditional thresholds of statistical significance.

**Table 4:** Adjusted OLS regression estimates for burnout at baseline on burnout at follow-up for groups of employees reporting a high versus a low degree of job resources

	High			Low			*p*-value for interaction^a^
Decision Latitude (*N* = 1169)							
	Unstandardized beta estimate	95% CI	*p*-value	Unstandardized beta estimate	95% CI	*p*-value	
Burnout baseline	0.58	0.50–0.66	< 0.001	0.64	0.58–0.71	< 0.001	0.09
Social Capital (*N* = 1081)							
	Beta	95% CI	*p*-value	Beta	95% CI	*p*-value	
Burnout baseline	0.59	0.52–0.67	< 0.001	0.67	0.60–0.74	< 0.001	0.13

Model adjusted for burnout at baseline, gender, age (continuous), level of education, marital status, children, and organization. Decision latitude models additional adjusted for social capital and social capital models additionally adjusted for decision latitude

Adjusted R^2^ = 0.378 for model with high decision latitude; 0.474 for model with low decision latitude; 0.391 for model with high social capital and 0.452 for model with low social capital

^a^The *p*-value for the interaction represents to *p*-value associated with the interaction between burnout and high/low decision latitude, or social capital, from a combined model

## Discussion

The aim of the present study was to longitudinally test the buffer-hypotheses of the Job Demand-Resources Model [14] and the Demand-Control Model [11].

We observed that psychological demands and burnout were associated with future burnout, measured 2 years later. However, the association between psychological demands and future symptoms of burnout was not attenuated by either social capital at work or decision latitude over this time period. These results suggest that the buffer-hypothesis should be rejected, and questions whether a high degree of job resources (specifically, decision latitude and social capital) can counteract the health-impairing effect of high job demands. The results confirm the few existing longitudinal studies on the subject [19–21], although in a larger and more heterogeneous sample, as well as a study focusing on other depressive symptoms, where the buffer hypothesis also was rejected [30]. In our study, burnout at baseline and follow-up were strongly correlated, showing that symptoms of burnout are likely to increase over time for employees who remain at their job, and confirming the enduring nature of burnout [5, 6] and highlighting the importance of targeting the adequate work conditions in order to stop its trajectory.

Taken together, these findings indicate that future research and practice cannot take for granted that job resources can buffer job demands, which needs to be taken into account both when designing studies and interventions. The results place psychological demands in the centre for the development of burnout and exhaustion and show the importance of reducing demands for prevention as well as for rehabilitation. Although job resources repeatedly have been found to increase work engagement [31], motivation and work enjoyment [32], the results of the current study suggest that in order to reduce burnout and exhaustion it is not enough to only increase opportunities for social interaction at work or providing the individual employee with increased autonomy or possibilities for skill use when psychological demands remain high. The results suggest that interventions need to focus on reducing psychological demands such as work pace, work load and the amount of conflicting demands, rather than focusing primarily on increasing job resources for workers in the hope that these will buffer the effects of demands on burnout.

While no differences were observed between the relationship between psychological demands and burnout among supportive versus unsupportive work environments, we did observe a stronger relationship between burnout at baseline and burnout at follow-up in environments with lower social capital and decision latitude. While this finding did not reach traditional levels of statistical significance (*p* < 0.05), this may be due to interaction analyses being underpowered in general [33]. This finding may suggest that opportunities to recover from burnout may be reduced in unsupportive work environments.

The results may especially have implications for managers and employers within public sector organizations, where the demands are frequently high and the decision latitude low. These organizations see an increasing number of employees that are exhausted and burned out, leading to complicated return to work processes [1, 34]. They are also under high pressure due to efficiency demands [35] which may increase the emotional and quantitative work load, work pace and conflicting demands such as those for productivity versus quality, or expectations from the management versus patients, clients or customers. Such pressures may increase sickness presenteeism, which is related to burnout and sick leave [36].

### Methodological considerations

Decision latitude and social capital have previously been found to predict work-related flow in the same sample [37] and are hence by definition job resources with buffering potentials [14]. It could however be argued that the hypothesis was rejected due to the variables that were used. Based on their review, Häusser et al. [18] suggest that in order for control or support to buffer the effect of high demands, these job resources must be based on qualitatively identical dimensions and measure the same aspect of work. In the present study, psychological demands concern the task level of work, while social capital at work concerns the collective dimension beyond the individual’s work tasks, reflecting the trust that is experienced between individuals at work and the collaborative capacities within groups [27, 32, 38]. Decision latitude and psychological demands are however both derived from the same instrument and are to be considered to concern the same task level of work as psychological demands [26].

A limitation of the study is that variables were only measured at two points in time. This constitutes a better test of the buffer hypothesis than cross-sectional studies and makes it possible to draw conclusions concerning across-time changes, but not concerning possible fluctuations in the variables between measurements [39]. Future studies should aim to replicate these findings with more measurement points. Further, it is possible that employees with increasing symptoms of burnout left their jobs, making the remaining sample more resilient to the development of burnout. We examined longitudinal attrition in the sample and observed that employees who did not respond to the follow-up survey did not differ for those that did in terms of burnout scores at baseline.

Another potential bias is negative affectivity, where conditions such as depression or neuroticism may impact reporting of personal resources or distress. This can be considered a common method bias, i.e., that the instrument itself causes variations in responses that does not correspond to actual differences. While this may be true for this study, the effect is somewhat limited by a longitudinal design, as outcomes at follow-up cannot influence factors at baseline.

The generalizability of the study is strengthened by the heterogeneous sample, containing respondents from seven organizations from various occupational groups and sectors, e.g., industry, health care, white collar workers, etc., at different levels in the organizations. This variation in the sample imply that there is a variety also in what demands and resources that the respondents meet in their work, which makes it more likely that the results are generalizable to a broader population, compared to studies focusing on single occupational groups. The mean values of psychological demands and burnout in this study are also similar to samples in other studies (cf. [2, 40–42]; cf. [43] for unstandardized measures from the same dataset as the current study). Women are over-represented in the data since the sample contains public organizations where women are in majority. It also reflects the gender distribution in sick leave due to burnout in Sweden where women are over-represented, although it has been noted that women and men with similar work conditions develop symptoms on burnout to the same extent [44]. Still, the over-representation may imply that the work conditions in female-dominated jobs dominates the results, which may limit the generalizability to male-dominated sectors. The drop-out rate at follow-up (36%) may further impact the generalizability of the findings. The dropout analysis shows that dropouts were slightly younger and had lower demands and decision latitude, while there were no significant differences for gender, burnout or social capital. This may imply that the follow-up results reflect slightly more demanding jobs with a higher amount of decision latitude, potentially limiting the generalizability to jobs with other work characteristics.

## Conclusions

This longitudinal study shows that higher levels of psychological demands are associated with higher levels of burnout 2 years later, and that this association is consistent across respondents who have higher and lower job resources such as decision latitude and social capital. The results have implications for rehabilitation and return to work as well as prevention, specifically related to the role of workplace adjustments and workplace supports that are required to prevent burnout at work. In order to prevent symptoms of burnout and the related sickness absence and to enable return to work after burnout or exhaustion, the present study shows the necessity to focus on job demands, e.g., reducing work pace, workload and conflicting demands at work, rather than only improving job resources.
